# Supplementary material for: A comparative study on folk sports inheritance in China and South Korea: Influencing factors and mediating roles
Source: PLoS One. 2025 Nov 14;20(11):e0336798. doi: 10.1371/journal.pone.0336798 (PMC12617914; doi:10.1371/journal.pone.0336798)
Supplement: Attachment — Submitted filename: Response to Reviewers.docx [file pone.0336798.s004.docx]

**Response to Reviewers**

Dear Editors and Reviewers,

We would like to express our sincere gratitude for the insightful comments and suggestions provided during the review of our manuscript. We have carefully addressed all of the points raised, and the revised manuscript has been updated accordingly. Below are our responses to the specific points raised by the Editors and Reviewers:

**Part 1: Response to the Editors**

1. Please ensure that your manuscript meets PLOS ONE's style requirements, including those for file naming.

Response: We have carefully revised the manuscript according to the PLOS ONE style guidelines. The manuscript and its accompanying files have been updated to match the required formatting. Specifically, we have followed the style templates provided at PLOS ONE Formatting Sample (Main Body) and PLOS ONE Formatting Sample (Title and Authors). Additionally, the file names have been adjusted as per the specified requirements.

2. Please include a complete copy of PLOS’ questionnaire on inclusivity in global research in your revised manuscript."

Response: We appreciate your suggestion and have completed the PLOS questionnaire on inclusivity in global research. The completed questionnaire has been uploaded as Supporting Information along with the revised manuscript.

3. Data Availability Statement: Please confirm whether or not your submission contains all raw data required to replicate the results of your study.

Response: Thank you for this comment. We have reviewed our manuscript and can confirm that the submission includes the minimal data set required for replication. This includes the values behind the means, standard deviations, and other reported measures, as well as the values used to build graphs and the points extracted from images for analysis. The relevant data are available in the Supporting Information files. Additionally, if needed, we are willing to deposit the data in a public repository and provide the relevant URLs, DOIs, or accession numbers. Please note that no ethical or legal restrictions prevent us from sharing this data.

4. Your ethics statement should only appear in the Methods section of your manuscript.

Response: We have relocated the ethics statement to the Methods section (3 Methods_3.3 Ethical Approval and Permissions) as per the guidelines. The statement has now been removed from all other sections of the manuscript.

**Part 2:** **Response to the Reviewers**

We would like to once again thank the reviewers for their insightful comments and suggestions. To ensure clarity and accuracy in our responses, the authors will address each of the reviewers' points individually in the table below.

| **Reviewer #1** | **Author's Revision** | **Author's Response** |
| --- | --- | --- |
| 1. Although relevant literature exists, the review could delve more deeply into the theoretical framework of intangible cultural heritage from anthropology or cultural studies. In this way, the research topic could be better understood. | The following content has been added to lines 151-168 of the manuscript.  At a deeper level, the theoretical interpretation of the influencing factors in folk sports inheritance is rooted in a multidisciplinary perspective. From the standpoint of intangible cultural heritage (ICH) studies, the inheritor is regarded as the creator and bearer of folk sports, referred to as the Inheritance Subject. The Inheritance Object denotes the intangible cultural heritage itself, which in this study refers specifically to distinct folk sports programs. Based on the theory of cultural tools, the Inheritance Intermediary functions as a bridge between the inheritance subject and the inheritance object, facilitating their interaction and encompassing elements such as awareness of protection and institutional mechanisms. From an anthropological perspective, the natural and social environment in which a sport exists plays a vital role in the development of ethnic sports; in this study, it is defined as the Inheritance Environment. From a management science perspective, the Inheritance Effect serves as a direct indicator and feedback mechanism for evaluating the effectiveness of cultural transmission, and is an essential component of the inheritance mechanism of traditional ethnic sports culture. Collectively, the five dimensions—Inheritance Subject, Inheritance Object, Inheritance Intermediary, Inheritance Environment, and Inheritance Effect—form a complete and dynamic coupling system that provides a theoretical lens for analyzing the differences in folk sports inheritance pathways between China and South Korea. | The theoretical foundation of the research on the influence of folk sports has been further enriched from multidisciplinary perspectives such as anthropology, cultural studies, management, and intangible cultural heritage studies. The additional content can be found in lines 151-168 of the manuscript. |
| 2. In the research design, it is more pertinent to put the sample or participants first to situate the reader the sample under study. | The revised table of contents is as follows.  3 Methods  3.1 Research Sample  3.2 Determination of Research Variables  3.3 Ethical Approval and Permissions  3.4 Survey Distribution and Evaluation  3.5 Data Analysis Methods  3.6 Inclusivity in Global Research | The research sample has been placed at the beginning of the Research Design section, with corresponding adjustments made to other content. |
| 3. p" in the level of significance is in italics, both at the foot of the tables and in the text. | The modifications can be found in lines 401, 407, 412, 421, 424, 436, 441, 452, and 456. | The revisions have been made. |
| 4. Statistical values should be without 0 before the comma, e.g., ** p < .01 Same in tables and text. | The modifications can be found in lines 401, 407, 412, 421, 424, 436, 441, 452, and 456. | The revisions have been made. |
| 5. Broader structural or historical factors that might explain the observed differences are not sufficiently discussed. For example, the text between lines 419 and 427, between 462 and 482 or between 485 and 509, do not have any citations to support the findings obtained, but are expressions to justify and argue the data. Therefore, they must rely on existing literature to contrast the empirical evidence obtained with previous studies. | The following references have been added:  10. Ma M, Wang DH. Research on influencing factors and optimized path of Yellow River basin folk sports inheritance based on grounded theory. Liaoning Sport Sci Technol. 2022;44(5):85–90,96. doi:10.13940/j.cnki.lntykj.2022.05.021.  36. Hong M, Kim J, Chang J, et al. DISSEMINATION OF DIGITAL HERITAGE USING MEDIA TECHNOLOGY-INTANGIBLE CULTURAL HERITAGE OF KOREA, DONGNAE CRANE DANCE[C]//DS 136: Proceedings of the Asia Design and Innovation Conference (ADIC) 2024. 2024: 335-342.  32. Sun JJ. Research on the coordinated development mechanism of ecological environment and sports culture: Taking China and South Korea as examples. Fresenius Environ Bull. 2021;(12):1–10.  38. Chen Y, Liu C. The cultural connotation of Gangneung Danoje Festival in South Korea. Cult Herit. 2015;(03):26–31.  39. Chen RY, Hu JF, Chen XR. Public law protection path of sports intangible cultural heritage from the perspective of comparative law. Bull Sport Sci Technol Lit. 2019;27(04):135–138. doi:10.19379/j.cnki.issn.1005-0256.2019.04.056.  40. Hu QS, Peng XY, He XR, et al. Spatial distribution characteristics and influencing factors of intangible cultural heritage inheritors in the Yangtze River Economic Belt. J Hunan Norm Univ (Nat Sci Ed). [Online ahead of print]:1–11. Available from: http://kns.cnki.net/kcms/detail/43.1542.N.20250605.1616.002.html.  41. Koo S. From Korea to Japan: A transnational perspective on South Korea's important intangible cultural properties and Zainichi Korean artists. Korean Stud. 2021;45(1):89–116.  42. Huang XT, Wang ZH, Zhang Y. Intergenerational transmission process and influencing factors of tourism livelihood in rural communities: A case study of Chongdu Village, Luanchuan County. Hum Geogr. 2023;38(01):158–168. doi:10.13959/j.issn.1003-2398.2023.01.018. | References have been added to strengthen the argument and provide a comparison with previous research. |
| 6. It is implicitly assumed that the proposed model is universal, without discussing its cultural limitations or its transferability to other contexts. | The following content has been added in lines 703-730 of the manuscript.  6.6 Future Directions  This research provides a theoretical foundation for understanding the inheritance mechanisms of folk sports in both China and South Korea, and it offers a potential reference framework for studies conducted in other countries and regions. However, it is important to acknowledge that the five-dimensional model proposed in this study—comprising Inheritance Subject, Inheritance Object, Inheritance Intermediary, Inheritance Environment, and Inheritance Effect—was developed based on the socio-cultural contexts of East Asia, particularly Confucian-influenced societies with shared traditions of collectivism, hierarchical structures, and government-led cultural protection. These cultural characteristics may not be equally present in other regions, which could limit the model’s direct applicability.  Future research should therefore avoid assuming the universality of this model and instead test its validity in diverse cultural contexts. Comparative studies involving countries with different historical trajectories, legal systems, and sport governance models (such as those in Europe, Africa, or Latin America) are needed to determine whether the model holds or requires adaptation. Such research could help identify culturally specific variables, uncover hidden assumptions embedded in the current framework, and refine the model for broader cross-cultural application.  Moreover, scholars should remain sensitive to the potential risks of cultural essentialism and ethnocentric bias when conducting international comparative research. It is equally important to consider the practical conditions and localized cultural traditions of each society when exploring feasible inheritance strategies. In the context of globalization, future work should also examine how transnational cultural flows, digital platforms, and evolving policy environments shape the dynamics of folk sports transmission, potentially transforming both the content and process of cultural inheritance. | The "Future Directions" section of the manuscript has been adjusted and expanded, with a discussion on its cultural limitations. |
| 7. The recommendations made are mostly one-sided (towards China) and no innovative ideas are offered for South Korea. | The following content has been added in lines 678-702 of the manuscript.  6.5 Recommendations for Improving Folk Sports Inheritance in South Korea  Although South Korea has established a relatively mature system for folk sports inheritance, there remains room for innovation and enhancement in the context of globalization, technological advancement, and intergenerational change. Based on the findings of this study, several targeted recommendations are proposed:  First, establish a dynamic assessment mechanism to identify and prioritize marginalized, endangered, or region-specific folk sports not yet included in the national intangible cultural heritage list. This should include regular evaluation protocols and differentiated resource allocation based on urgency levels and regional significance.  Second, leverage South Korea's technological expertise to create innovative digital preservation and transmission platforms. This includes developing VR-based experiential learning environments, AI-assisted cultural documentation systems, and interactive mobile applications that can engage younger demographics while maintaining cultural authenticity.  Third, transition from top-down institutional preservation toward community-driven revitalization models. This involves establishing participatory governance structures that empower local communities, grassroots organizations, and youth groups to take ownership of folk sports transmission, ensuring cultural practices remain living traditions rather than static exhibits.  Finally, capitalize on South Korea's global cultural influence through strategic integration of folk sports into existing cultural export channels. This includes embedding traditional sports elements into K-culture content, establishing international folk sports festivals, and developing educational exchange programs that position South Korea as a leader in innovative cultural heritage preservation. | A new section, "6.5 Recommendations for Improving Folk Sports Inheritance in South Korea," has been added to address and correct the limitation of previously providing recommendations solely for China. |
| 8. I invite the authors to use other citations to support the statements made in the text, since their bibliographic references, in their entirety, are centered on authors from the Asian continent. In this way, it is possible to enrich the perspective and the variables under study of other citizens at the international level | The following references have been added:  27. Wertsch JV. Mind as action. New York: Oxford University Press; 1998.  32. Sun JJ. Research on the coordinated development mechanism of ecological environment and sports culture: Taking China and South Korea as examples. Fresenius Environ Bull. 2021;(12):1–10.  36. Hong M, Kim J, Chang J, et al. Dissemination of digital heritage using media technology—intangible cultural heritage of Korea, Dongnae Crane Dance. In: DS 136: Proc Asia Des Innov Conf (ADIC) 2024. 2024:335–342.  41. Koo S. From Korea to Japan: A transnational perspective on South Korea's important intangible cultural properties and Zainichi Korean artists. Korean Stud. 2021;45(1):89–116.  43. Kurin R. Safeguarding intangible cultural heritage in the 2003 UNESCO Convention: A critical appraisal. Mus Int. 2004;56(1–2):66–77. | The sources of the references have been diversified, with the addition of references that offer a broader international perspective. |

| **Reviewer #2** | **Author's Revision** | **Author's Response** |
| --- | --- | --- |
| 1. Theoretical Clarity: The conceptual framework requires stronger grounding in relevant literature. Please clarify how the five dimensions relate to existing theories of cultural transmission, sports sociology, or heritage studies. | The following content has been added to lines 151-168 of the manuscript.  At a deeper level, the theoretical interpretation of the influencing factors in folk sports inheritance is rooted in a multidisciplinary perspective. From the standpoint of intangible cultural heritage (ICH) studies, the inheritor is regarded as the creator and bearer of folk sports, referred to as the Inheritance Subject. The Inheritance Object denotes the intangible cultural heritage itself, which in this study refers specifically to distinct folk sports programs. Based on the theory of cultural tools, the Inheritance Intermediary functions as a bridge between the inheritance subject and the inheritance object, facilitating their interaction and encompassing elements such as awareness of protection and institutional mechanisms. From an anthropological perspective, the natural and social environment in which a sport exists plays a vital role in the development of ethnic sports; in this study, it is defined as the Inheritance Environment. From a management science perspective, the Inheritance Effect serves as a direct indicator and feedback mechanism for evaluating the effectiveness of cultural transmission, and is an essential component of the inheritance mechanism of traditional ethnic sports culture. Collectively, the five dimensions—Inheritance Subject, Inheritance Object, Inheritance Intermediary, Inheritance Environment, and Inheritance Effect—form a complete and dynamic coupling system that provides a theoretical lens for analyzing the differences in folk sports inheritance pathways between China and South Korea. | The theoretical foundation of the research on the influence of folk sports has been further enriched from multidisciplinary perspectives such as anthropology, cultural studies, management, and intangible cultural heritage studies. The additional content can be found in lines 151-168 of the manuscript. |
| 2. Statistical Reporting: While the use of SEM is appropriate, key statistical details—such as model fit indices and assumption testing—are missing. Please report these clearly and transparently. | The content of this section can be found in Tables 3, 5, and 6 of the manuscript. | The author has re-examined the manuscript and confirmed that it presents the key model fit indices and assumption testing. |
| 3. Data Availability: In line with journal policy, the dataset underlying your analysis must be made fully accessible, either through a public repository or supplementary files. | The original data for all research samples involved in this study have been uploaded to the Supplementary Files. | The original data have been uploaded to the Supplementary Files. |
| 4. Language and Presentation: The manuscript contains numerous grammatical, structural, and stylistic issues. We strongly recommend professional English language editing to ensure clarity and consistency. |  | Thank you for your valuable feedback. We apologize for the language issues in our manuscript. We have worked on the manuscript for a long time, and the repeated addition and removal of sentences and sections has unfortunately impacted its readability. We have now made revisions to both the language and readability, and have involved native English speakers for language corrections. We sincerely hope that the flow and language quality have been significantly improved. |
| 5. Policy Implications: The practical contribution of your findings could be strengthened. Consider elaborating on how your model informs institutional practice or bilateral collaboration in cultural preservation. | The following content has been added in lines 628-646 of the manuscript.  6.3 Policy Contributions  In addition to its theoretical innovations, this study provides valuable policy implications for cultural heritage governance at both the national and bilateral levels.  First, the five-dimensional model developed in this research offers a practical diagnostic tool for cultural institutions to evaluate their folk sports transmission systems. By identifying structural imbalances—such as an overemphasis on material documentation or underdeveloped intermediary mechanisms—this model can inform more targeted and holistic strategies in policy formulation.  Second, the model provides a basis for improving institutional practices. It suggests that cultural heritage protection should go beyond object-centered approaches and instead invest in cultivating local leadership (inheritance subject), building community-based transmission platforms (inheritance intermediary), and creating more integrated policy environments (inheritance environment). These insights can guide cultural bureaus, heritage centers, and educational institutions in enhancing the sustainability of folk sports practices.  Third, the model opens up new pathways for bilateral cooperation between China and South Korea. Shared inventories of folk sports heritage, joint training programs for youth practitioners, and cross-border evaluation standards based on the five dimensions can foster mutual learning, strategic alignment, and long-term cultural exchange. | A new section, "Policy Contributions," has been added in Section 6.3, where we systematically elaborate on how the five-dimensional model proposed in this study can provide evaluation and intervention pathways for cultural institutions. Additionally, the specific applications of this model in the collaboration of folk sports between China and South Korea are discussed. |

We hope that the revisions meet the expectations of the reviewers and editors. Please feel free to reach out if further clarification is required. Thank you again for your valuable feedback.

Sincerely,

Yu Hou
