# Supplementary material for: A comparative study on folk sports inheritance in China and South Korea: Influencing factors and mediating roles
Source: PLoS One. 2025 Nov 14;20(11):e0336798. doi: 10.1371/journal.pone.0336798 (PMC12617914; doi:10.1371/journal.pone.0336798)
Supplement: Attachment — Submitted filename: Response to Reviewers-250909.docx [file pone.0336798.s005.docx]

**Response to Reviewers**

Dear Editors and Reviewers,

We sincerely thank the Editors and Reviewers for their careful evaluation, insightful comments, and constructive suggestions, as well as for the time and effort devoted to improving our manuscript. We have carefully addressed all the points raised, and the revised manuscript has been updated accordingly. Below, we provide detailed, point-by-point responses to the editors’ and reviewers’ concerns, specifying how each has been addressed in the revised manuscript.

**Response to Editor：**

**Comment 1:** Please ensure that you refer to Figure 1 in your text as, if accepted, production will need this reference to link the reader to the figure.

**Response 1:**

We appreciate the reviewer’s attention to this detail. We have now explicitly referred to *Figure 1* in the text (lines 202-203, Section 2) to ensure proper linkage during production.

**Comment 2:** Please provide additional details regarding participant consent. In the Methods section, please ensure that you have specified (1) whether consent was informed and (2) what type you obtained (for instance, written or verbal). If your study included minors, state whether you obtained consent from parents or guardians. If the need for consent was waived by the ethics committee, please include this information.

**Response 2:**

Thank you for this important suggestion. We have revised the description of participant consent in the Methods section (lines 268-273, Section 3.3). We now clarify that informed electronic consent was obtained from all participants in written form via the online platforms (Wenjuanxing in China and Google Forms in South Korea). Participants confirmed that they had read and understood the study purpose, the voluntary nature of participation, and the confidentiality of their responses. We also explicitly state that the study did not involve minors, and no waivers of consent were granted by the ethics committee.

**Response to Reviewer #1:**

We sincerely thank Reviewer #1 for the positive evaluation of our revised manuscript. We greatly appreciate your recognition of the improvements made in response to previous comments and suggestions. Your feedback confirms that the revisions have enhanced the quality of the manuscript and ensured compliance with the guidelines and ethical standards of *PLOS ONE*.

**Response to Reviewer #2:**

We sincerely thank Reviewer #2 for the detailed and constructive feedback on our revised manuscript. We greatly appreciate your recognition of the improvements made in figure captions, literature engagement, and the overall empirical approach. Below, we provide point-by-point responses to your specific concerns.

**Comment 1:** While the introduction has been refined since the initial submission, it still lacks a sharply defined research gap and explicit statement of novelty relative to prior studies.

**Response 1:**

In the revised Introduction (lines 60–69, 93–103), we explicitly addressed this concern by: (1) identifying three specific research gaps—namely, the predominant reliance on qualitative methodologies, the scarcity of cross-national comparative studies, and the absence of a unified analytical framework; (2) linking each gap directly to the corresponding contributions of this study; and (3) highlighting the novelty of introducing and empirically validating a five-dimensional framework using cross-national data. These revisions aim to clearly delineate the study’s rationale and scholarly contribution.

**Comment 2:** The link between the literature review and the specific aims of this paper is not as strong as it could be, especially in justifying why this study is needed now.

**Response 2:**

In the revised manuscript (lines 174–186), we strengthened the link between the literature review and the research aims by emphasizing the timeliness of the study. Specifically, China’s rapid modernization is accelerating the decline of traditional folk sports, whereas Korea’s institutionalized preservation through UNESCO recognition and legal frameworks provides a mature model. This contrast highlights the urgency of conducting a cross-national comparison. Moreover, the identified gaps—namely, the reliance on qualitative studies, the absence of cross-national frameworks, and the lack of unified evaluation indices—are now directly linked to our proposed quantitative, five-dimensional approach, clarifying the necessity and relevance of the present study.

**Comment 3:** Although some methodological clarifications were made since the last submission, key details are still missing, including sampling procedures, justification for variable inclusion/exclusion, and handling of missing data.

**Response 3:**

In the revised manuscript (lines 222–233, Section 3.1: Research Sample), we thank the reviewer for emphasizing the need for methodological clarity. We have added a detailed description of our stratified sampling procedure across stakeholder groups in both countries, as well as the rationale for inclusion and exclusion of variables based on prior theoretical models and pilot testing. Additionally, all questionnaires were fully completed, resulting in no missing data, and thus no imputation procedures were required. These revisions enhance transparency and ensure methodological rigor, consistent with best practices for global and cross-cultural research.

**Comment 4:** While results tables and figures are now clearer, effect sizes are still inconsistently reported alongside p-values.

**Response 4:**

In the revised manuscript (lines 415–422, Section 4.3: Empirical Results), we updated Tables 6 and 7 to consistently report standardized effect sizes (β coefficients) alongside p-values and their 95% confidence intervals. For mediation analyses, standardized indirect effects and bias-corrected bootstrap confidence intervals were also included. These revisions improve the interpretability of our results by balancing statistical significance with substantive effect size and ensure alignment with standard reporting practices in structural equation modeling (SEM).

**Comment 5:** The narrative continues to repeat table content rather than interpret it.

**Response 5:**

In the revised manuscript (lines 431–479, 489–498), Sections 4.3.1–4.3.5 were carefully revised to minimize redundant repetition of tabulated data and to strengthen interpretative commentary. Rather than restating coefficients and test statistics already presented in the tables, the text emphasizes observed differences between China and South Korea, highlights underlying mechanisms (e.g., cultural identity versus institutional reinforcement), and draws comparative insights. Each subsection concludes with a concise synthesis that underscores key implications while avoiding over-generalization.

**Comment 6:** Although expanded, the discussion still tends to summarise rather than critically engage with the literature. Also, over-generalisation remains a concern; conclusions should be more closely tied to the scope of the sample and study design.

**Response 6:**

In the revised manuscript (lines 503–606, Sections 5.1–5.6), we substantially refined the discussion to enhance interpretative depth and scholarly engagement. Rather than reiterating tabulated results, each subsection emphasizes explanatory interpretation, cross-national contrasts, and theoretical implications. For instance, the role of the Inheritance Object as a cultural anchor in China versus an institutional component in Korea is highlighted in relation to Jiang[30] and Kurin[39]. Recent studies (e.g., Hong et al.[34]; Chen & Liu[36]; Sun[38]) are integrated to critically examine how intermediaries and environments function as multiplicative factors within institutional systems. We explicitly connect effect size differences (e.g., β values for China vs. Korea) to systemic contrasts, while engaging with comparative frameworks from Cui et al.[9] and Koo[42]. These revisions reduce redundancy, strengthen scholarly dialogue, and more firmly situate our findings within existing debates, providing a clearer and more focused presentation of the study’s contributions.

We hope that these revisions meet the expectations of the reviewers and editors. Please do not hesitate to contact us if further clarification is required. Thank you again for your valuable feedback.

Sincerely,

Yu Hou
